# Orthogeriatrics prevents functional decline in hip fracture patients: report from two randomized controlled trials

**DOI:** 10.1186/s12877-021-02152-7

**Published:** 2021-03-25

**Authors:** Shams Dakhil, Pernille Thingstad, Frede Frihagen, Lars Gunnar Johnsen, Stian Lydersen, Eva Skovlund, Torgeir Bruun Wyller, Olav Sletvold, Ingvild Saltvedt, Leiv Otto Watne

**Affiliations:** 1 Department of Geriatric Medicine, Oslo University Hospital, Oslo, Norway; 2 Institute of Clinical Medicine, University of Oslo, Oslo, Norway; 3 Department of Neuromedicine and Movement Science, Norwegian University of Science and Technology (NTNU), Trondheim, Norway; 4 Division of Orthopedic Surgery, Oslo University Hospital, Oslo, Norway; 5 Orthopedic Trauma Unit, Department of Orthopedic Surgery, St. Olavs Hospital, Trondheim University Hospital, Trondheim, Norway; 6 Norwegian National Advisory Unit on Trauma, Division of Emergencies and Critical Care, Oslo University Hospital, Oslo, Norway; 7 Department of Mental Health, Regional Centre for Child and Youth Mental Health and Child Welfare, Norwegian University of Science and Technology (NTNU), Trondheim, Norway; 8 Department of Public Health and Nursing, Norwegian University of Science and Technology (NTNU), Trondheim, Norway; 9 Department of Geriatrics, St. Olavs Hospital, Trondheim University Hospital, Trondheim, Norway

**Keywords:** Orthogeriatric, Hip fracture, Activities of Daily living (ADL)

## Abstract

**Background:**

The incidence of hip fractures are expected to increase in the following years. Hip fracture patients have in addition to their fracture often complex medical problems, which constitute a substantial burden on society and health care systems. It is thus important to optimize the treatment of these patients to reduce negative outcomes. The aim of this study was to assess the effect of comprehensive orthogeriatric care (CGC) on basic and instrumental activities of daily living (B-ADL and I-ADL).

**Methods:**

This study is based on two randomized controlled trials; the Oslo Orthogeriatric Trial and the Trondheim Hip Fracture Trial. The two studies were planned in concert, and data were pooled and analyzed using linear mixed models. I-ADL function was assessed by the Nottingham Extended ADL Scale (NEADL) and B-ADL by the Barthel ADL (BADL) at four and twelve months after surgery.

**Results:**

Seven hundred twenty-six patients were included in the combined database, of which 365 patients received OC and 361 patients received CGC. For the primary endpoint, I-ADL at four months was better in the CGC group, with a between-group difference of 3.56 points (95 % CI 0.93 to 6.20, *p* = 0.008). The between-group difference at 12 months was 4.28 points (95 % CI 1.57 to 7.00, *p* = 0.002). For B-ADL, between-group difference scores were only statistically significant at 12 months. When excluding the patients living at a nursing home at admission, both I-ADL and B-ADL function was significantly better in the CGC group compared to the OC group at all time points.

**Conclusions:**

Merged data of two randomized controlled trials showed that admitting hip fracture patients to an orthogeriatric care unit directly from the emergency department had a positive effect on ADL up to twelve months after surgery.

## Background

Patients suffering from a hip fracture are often frail; suffering multiple comorbidities, and are often subjected to polypharmacy [1]. The prefracture functional level of hip fracture patients has been found to be a strong and consistent predictor of short- and long-term rehabilitation outcome [2]. Only one third of patients return to their prefracture function, and one third will require further nursing home care [3]. Since the incidence is expected to increase, hip fractures will become a progressively larger public health burden [4–6].

Hip fracture patients are a large and resource-demanding group. Several studies have shown that orthogeriatric care is beneficial regarding length of stay in hospital, waiting time to surgery, fewer surgical and medical complications and survival [7–15]. There are several different orthogeriatric models; ranging from orthopedic wards with a geriatric consultant service to an integrated care ward [7]. However, due to the heterogeneity of the different studies both in measured outcomes and study design, it is challenging to draw conclusions on what type of orthogeriatric care model is superior. In addition, most studies have evaluated the effect based on register data (mortality, length of stay, re-admissions) and very few have assessed the effect based on a face-to-face evaluation of the patients in the months following discharge.

It has been argued that hip fracture patients benefit from an admission to a geriatric ward instead of an orthopedic ward [8, 16–19]. In such a model, “Geriatric and rehabilitation ward and orthopedic consultant service” according to Kammerlander [7], the patient is admitted directly from the emergency department to the geriatric ward. The patient has the entire stay (except for surgery) in the geriatric ward, and the orthopedics serve as consultants. Several studies have evaluated the effect of the implementation of such a model and the overall impression is that it is beneficial [20–24]. However, due to the heterogeneity in study design and outcomes, there is a need for multi-center studies which will allow for increased generalizability and give more precise estimates of the effect of such models.

Recently there have been two randomized controlled trials (RCTs) in Norway assessing the effect of this model; The Trondheim Hip Fracture Trial [25] and the Oslo Orthogeriatric Trial [1]. In both studies, the control group received traditional orthopedic care. The Oslo and Trondheim studies were planned in concert, and we have now merged data from these studies. This pooled data set will yield information from a larger and more heterogeneous group of hip fracture patients and increased statistical power will give more precise estimates of the effect of the model. The aim of the current study was to assess the effect of our orthogeriatric model on Activities of Daily Living (ADL) – both instrumental ADL (I-ADL) and basic ADL (B-ADL) - four and twelve months after surgery.

## Methods

 Inclusion and randomization took place in the emergency department in the respective hospitals in both trials. In Oslo randomization was based on computer-generated random numbers (blocks of variable and unknown size) and was carried out by a statistician not involved in the clinical service. Randomization was also stratified according to whether or not the patients were admitted from nursing homes. In Trondheim patients were randomly assigned in a 1:1 ratio by a nurse. In both hospitals patients were transferred to the allocated wards directly from the emergency department. The intervention group received a CGC service preoperatively as well as postoperatively. Surgical and anesthesiologic procedures were similar in both groups. Four- and twelve-month follow-up assessments were carried out at the hospital by study nurses blinded to group allocation. If the patients were unable to visit the hospitals the study nurses visited the patients where they were living at the specific time point and conducted the follow-up interview face to face. Since the intervention was at ward level, data collection during the index stay could not be blinded.

### Oslo orthogeriatric trial

Recruitment lasted from September 2009 to January 2012 at Oslo University Hospital. All hip fracture patients were eligible for the trial, unless if the fracture was due to a high-energy trauma or if the patient was moribund at admission. Both home-dwelling patients and patients living in a nursing home at admission, at all ages were included, in total 329 patients [1].

Patients randomized to intervention were treated in the acute geriatric ward; both pre- and postoperatively. A team consisting of a geriatrician, nurse, physiotherapist and occupational therapist were responsible for delivering the CGC service. They were expected to assess patients during their first day on the ward, as well as conducting daily meetings to coordinate treatment and to plan discharge. The CGC service included medication reviews, early and intensive mobilization, optimizing pre- and postoperative nutrition and early discharge planning. Details about the clinical routines have been published [26].

The primary outcome for this study was cognitive function four months after surgery, and the secondary outcomes included delirium, delirium severity, length of stay, mortality, mobility, place of residence, Instrumental (I-ADL) and basic (B-ADL) function, and weight changes. The intervention had no impact on the primary outcome. However, better mobility (measured by the Short Physical Performance Battery (SPPB [27]) was found in home-dwelling patients [1].

### Trondheim hip fracture trial

Recruitment lasted from April 2008 to December 2010 at St. Olavs Hospital, Trondheim University hospital. All home-dwelling patients above the age of 70, and who were able to walk 10 m or more before the fracture were included (n = 397). Patients that had suffered a pathological fracture, undergone multiple traumas, or had a short life expectancy, as well as patients already living in a nursing home were excluded [25].

Patients randomized to intervention were treated in the geriatric ward with CGC service; both pre- and postoperatively. The CGC service included comprehensive medical assessment and treatment, early rehabilitation and early planning of discharge. Details about the clinical routines have been published [28].

For this study the primary outcome was mobility after four months measured by the SPPB, and secondary outcomes included I-ADL, B-ADL, cognition, quality of life, fear of falling, depression, gait control and daily physical activity. The study found a positive effect of the intervention on the primary outcome, and also on several of the secondary outcomes (I-ADL, B-ADL, fear of falling, quality of life, gait control and daily physical activity) [25].

### TOO HIP (the OslO and Trondheim HIP fracture trial) database

**Fig. 1:**
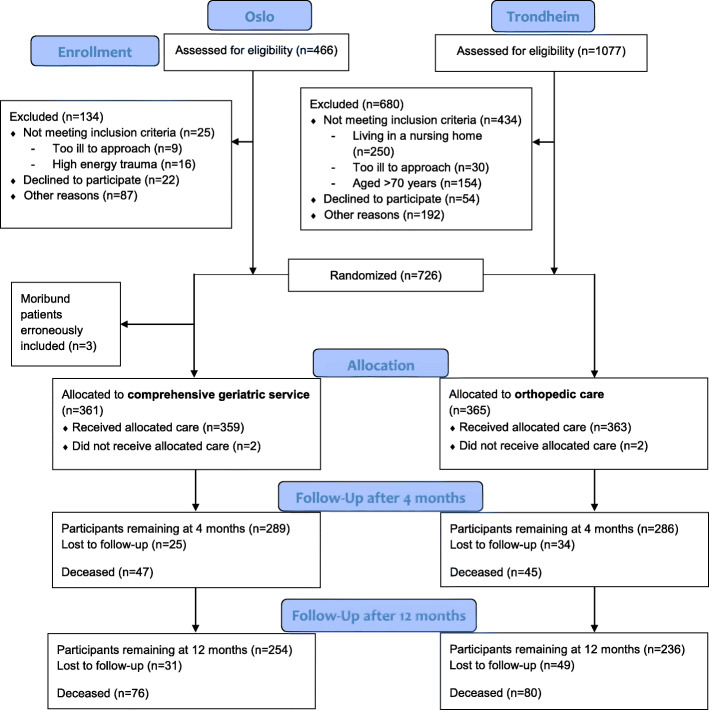
Consort flow diagram

The Trondheim Hip Fracture Trial and the Oslo Orthogeriatrics Trial were planned in concert, and similar design and outcomes were chosen for future pooling of data as described in their protocols [26, 29]. The goal was to make a larger and more heterogeneous database to provide the opportunity for more precise estimates on outcomes (Fig. 1). For assessing the effect of intervention on I-ADL and B-ADL function in the combined dataset, Nottingham Extended Activities of Daily Living Scale (NEADL) (range 0–66, higher scores indicate better function) [30] four months after surgery was chosen as the primary outcome. Secondary outcomes included NEADL at twelve months postoperatively, The Barthel ADL Index (BADL) (measures degree of independence in ten basic ADL functions (range 0–20), higher scores indicate better function) [31] score at four and twelve months postoperatively, intra-hospital mortality and cumulative mortality at four and twelve months postoperatively, and new nursing home admissions.

### Statistical methods

A statistical analysis plan was completed prior to any analyses [32]. The primary efficacy analysis was carried out using linear mixed model with NEADL as dependent variable, patient as random factor, time point (baseline, four months and twelve months) as fixed factor, and treatment group, site (Oslo versus Trondheim), age, sex, fracture type (extracapsular versus intracapsular), dwelling at home (versus nursing home), and the interaction between time points after inclusion and treatment group as covariates. Similar mixed model analyses were carried out with BADL score as the dependent variable. Dichotomous outcomes were analysed unadjusted, comparing proportions in the two treatment groups. In addition, they were compared using logistic regression, unadjusted, and adjusted for site, age, sex, fracture type and dwelling at home.

A two-sided p-value below 0.05 was taken as an indicator of statistical significance, and 95 % confidence intervals (CI) are reported where relevant. Missing items within the NEADL and BADL scales were imputed by the mean score for the remaining items that were answered, if at least 80 % of the items on the scale were answered. Normality of residuals was checked by visual inspection of Q-Q-plots. All statistical analyses were done using IBM SPSS statistics 22–25.

## Results

In total 726 patients were included in the combined database, of which 365 patients received traditional OC and 361 patients received CGC. Baseline characteristics did not differ between the groups (Table 1). Mean age was 83.0 years (SD 7.7), 542 (74.7 %) were women, and 102 (14.0 %) were living in a nursing home at admission. The groups were similar in function as measured by NEADL and BADL at baseline.

**Table 1:** Baseline characteristics

	Comprehensive geriatric care (*N* = 361)	Orthopedic care (*N* = 365)
Age, mean (SD)	83.0 (7.3)	83.0 (8.0)
Male (%)	95 (26.3)	89 (24.4)
Living in a nursing home at admission (%)^a^	52 (14.4)	50 (13.7)
Barthel Index, mean (SD)^b^	17.2 (3.7)	17.4 (3.6)
NEADL, mean (SD)^c^	37.1 (20.6)	37.5 (19.9)
Type of fracture		
- Extracapsular (%)	144 (39.9)	141 (38.6)
- Intracapsular (%)	217 (60.1)	224 (61.4)
Surgical treatment^d^		
- Hemiarthroplasty (%)	148 (41.2)	155 (42.8)
- Osteosynthesis (%)	208 (57.95)	199 (55.0)
- Total hip replacement (%)	2 (0.6)	5 (1.4)
- Girdlestone (%)	1 (0.3)	0 (0)
- Not operated (%)	0 (0)	3 (0.8)
- Died before surgery	2 (0.6)	3 (0.8)
Injury occurred indoors (%)^e^	270 (77.6)	279 (78.8)

*SD *standard deviation, *Barthel Index *Barthel Index for Activities of Daily Living, *NEADL *Nottingham Extended Activities of Daily Living

^a^Patients admitted from nursing homes were excluded in Trondheim

^b^Barthel Index was missing from 10 in the orthopedic care group and 6 patients in the comprehensive geriatric care group

^c^NEADL was missing from 12 patients in the orthopedic care group and 9 patients in the comprehensive geriatric care group

^d^Information about surgical treatment was missing/unknown in 3 patients in the orthopedic care group and 2 patients in the comprehensive geriatric care group

^e^Information about where the injury occurred (inside/outside) was unknown in 11 patients in the orthopedic care group and 13 patients in the comprehensive geriatric care group

At four months the CGC group had better mean NEADL scores than the OC group with a between-group difference of 3.56 points (CI 0.93 to 6.20, *p* = 0.008; Table 2). The between-group difference at twelve months was 4.28 points (CI 1.57 to 7.00, *p* = 0.002; Table 2).

**Table 2:** Linear mixed model with NEADL and Barthel Index

	Comprehensive geriatric care		Orthopedic care		Difference	
	**N**	**Mean (SE)**	**N**	**Mean (SE)**	**Estimate (95 % CI)**	***p***-value
**4 months**	295		291			
NEADL^a^	281	30.34 (0.95)	276	26.77 (0.95)	3.56 (0.93 to 6.20)	0.008
Barthel Index^b^	286	15.44 (0.22)	284	15.09 (0.22)	0.34 (-0.25 to 0.94)	0.26
**12 months**	260		245			
NEADL^c^	253	30.59 (0.97)	234	26.31 (0.99)	4.28 (1.57 to 7.00)	0.002
Barthel Index^d^	251	15.46 (0.22)	234	14.78 (0.23)	0.68 (0.05 to 1.31)	0.034

Linear mixed model with NEADL and Barthel Index, respectively, as dependent variable, patient as random factor, time point (baseline, 4 months and 12 months after surgery) as fixed factor, and treatment group, site (Oslo versus Trondheim), age, sex, fracture type, dwelling at home (versus nursing home), and the interaction between time and treatment as covariates

*SE *standard error, *CI *confidence interval, *NEADL *Nottingham Extended Activities of Daily Living scale, *Barthel Index *Barthel Activities of Daily Living index

^a^NEADL at 4 months missing from 15 patients in the orthopedic care group and 14 patients in the comprehensive geriatric care group

^b^Barthel Index at 4 months missing from 7 patients in the orthopedic care group and from 9 patients in the comprehensive geriatric care group

^c^NEADL at 12 months missing from 11 patients in the orthopedic care group and 7 patients in the comprehensive geriatric care group

^d^Barthel Index at 12 months missing from 11 patients in the orthopedic care group and missing 9 patients in the comprehensive geriatric care group

For BADL; between-group difference scores were in favor of CGC on four and twelve months, but were only statistically significant at 12 months (4 month: between-group difference at 0.34 and CI 0.25 to 0.94, *p* = 0.26, and 12 months: between-group difference at 0.68 and CI 0.05 to 1.31, *p* = 0.034; Table 2).

When excluding the patients living at a nursing home at baseline, the ADL function was better in the intervention group at all time points; both for NEADL (4 months: between-group difference at 4.56 and CI 1.61 to 7.52, *p* = 0.003 and twelve months: between-group difference at 5.41 and CI 2.38 to 8.44, *p* < 0.001; Table 3) and for BADL (four months: between-group difference at 0.67 and CI 0.06 to 1.28, *p* = 0.030 and twelve months: between-group difference at 0.97 and CI 0.34 to 1.60, *p* = 0.003; Table 3).

**Table 3:** ADL excluding nursing home patients

	Comprehensive geriatric care		Orthopedic care		Difference	
	**N**	**Mean (SE)**	**N**	**Mean (SE)**	**Estimate (95 % CI)**	*p*-value
**4 months**	260		253			
NEADL^a^	247	33.88 (1.06)	241	29.31 (1.07)	4.56 (1.61 to 7.52)	0.003
Barthel Index^b^	251	16.54 (0.22)	247	15.87 (0.22)	0.67 (0.06 to 1.28)	0.030
**12 months**	234		217			
NEADL^c^	227	34.33 (1.08)	208	28.92 (1.10)	5.41 (2.38 to 8.44)	< 0.001
Barthel Index^d^	226	16.59 (0.23)	207	15.62 (0.23)	0.97 (0.34 to 1.60)	0.003

Linear mixed model with NEADL and Barthel Index, respectively, as dependent variable, patient as random factor, time point (baseline, 4 months and 12 months after surgery) as fixed factor, and treatment group, site (Oslo versus Trondheim), age, sex, fracture type, dwelling at home (versus nursing home), and the interaction between time and treatment as covariates

*SE *standard error, *95 % CI *95 % confidence interval, *NEADL *Nottingham Extended Activities of Daily Living scale, *Barthel Index *Barthel Activities of Daily Living index

^a^NEADL at 4 months missing from 12 patients in the orthopedic care group and 13 patients in the comprehensive geriatric care group

^b^Barthel Index at 4 months missing from 6 patients in the orthopedic care group and from 9 patients in the comprehensive geriatric care group

^c^NEADL at 12 months missing from 9 patients in the orthopedic care group and 7 patients in the comprehensive geriatric care group

^d^Barthel Index at 12 months missing from 10 patients in the orthopedic care group and missing 8 patients in the comprehensive geriatric care group

The mean preoperative waiting time was not different between groups (30.5 vs. 29.2 h, *p* = 0.76; Table 4). Length of hospital stay was longer in the CGC group (mean 12.8 vs. 9.8 days *p* < 0.001; Table 4). In-hospital mortality was the same between the groups (2.2 vs. 2.2 %, *p* = 0.98; Table 4). Also, there was no significant difference in number of deaths at 4 months (13.0 vs. 12.3 %, *p* = 0.78) or 12 months (20.8 vs. 21.6 %, *p* = 0.78) after surgery. There was a trend towards fewer new nursing home admissions in the CGC group at 4 months (16.9 vs. 20.9 %, *p* = 0.23) and 12 months (19.2 vs. 25.3 %, *p* = 0.11; Table 4).

**Table 4:** Impact of intervention during hospital stay, and 4 months and 12 months after hospital stay

**Hospital stay**	**Comprehensive geriatric care (*N*** **=** **361)**	**Orthopedic care (*N*** **=** **365)**	***p*-value**
Waiting time for surgery in hours, mean (SD)^a^	30.5 (26.8)	29.2 (19.1)	0.76^1^
Length of stay in days, mean (SD)	12.8 (7.9)	9.8 (6.7)	< 0.001^1^
In-hospital mortality (%)	8 (2.2)	8 (2.2)	0.98^2^
**4 months after surgery**	**Comprehensive geriatric care** **(*N* = 295)**	**Orthopedic care** **(*N* = 291)**	
New nursing home admissions (%)^b^	44 (16.9)	53 (20.9)	0.23^2^
**12 months after surgery**	**Comprehensive geriatric care** **(*N* = 260)**	**Orthopedic care** **(*N* = 245)**	
New nursing home admissions (%)^b^	45 (19.2)	55 (25.3)	0.11^2^

*SD *standard deviation

^a^Waiting time for surgery in hours, defined as hours from admission to start of anesthesia, missing from 7 patients in the orthopaedic care group and 2 patients in the comprehensive geriatric care group

^b^Information about new nursing home admissions missing/unknown in 2 patients in the orthopedic care group at 4 months, and 1 patient in the orthopedic care group at 12 months. Fifty patients from the orthopedic care group and fifty-two patients from the geriatric care group lived in a nursing home before the hip fracture

^1^Mann-Whitney U Test

^2^Pearson Chi-Square test

## Discussion

The present study merged data from two Norwegian RCTs evaluating impact of CGC performed in acute geriatric wards compared to usual care in orthopaedic wards in treatment of hip-fracture patients. Our main result is that I-ADL was better in hip fracture patients treated with CGC as compared to usual care four and twelve months post-operatively. B-ADL as well, was better in the intervention group after twelve months. The effect of intervention on I-ADL and B-ADL was stronger when excluding patients admitted from a nursing home. A difference of 2.4 points on NEADL is considered to be clinically significant [33] and one point on BADL is the difference between being independent or not in basic ADL functions (walking, feeding, toilet use etc.). We therefore believe that the effects we find in our study is clinically relevant.

Our findings are in line with other studies conducted on a similar orthogeriatric care model as ours. In a quasi-RCT, Adunsky et al. showed that patients allocated to the intervention arm had almost a two-fold chance of successful rehabilitation outcome defined as more than 50 % increase in “relative functional gain” [23]. Stenvall et al., conducted a prospective RCT and showed that significantly more patients allocated to intervention had regained independence in both I-ADL and B-ADL performance both four and twelve months after surgery, measured by the Katz Index of Independence in ADL [24]. To our knowledge these are the only other studies conducted in a geriatric ward with ADL as an end point. Other studies conducted in an orthopedic ward with varying geriatric liaison service have also evaluated the effect of intervention on ADL; some have shown an effect of intervention [9, 19, 34–37], while others have only shown a trend [38] or no effect [39, 40].

The mean length of hospital stay was significantly longer in the intervention group in our study. A reduction of length of stay is often considered cost-effective [41–44]. However, in addition to costs of the initial hospitalization there are several other aspects, such as re-admissions and need of rehabilitation and nursing homes. If longer length of stay results in increased ADL function it might therefore be beneficial for the society in the long run, as was also the conclusion in the Trondheim Hip Fracture Trial that calculated the full cost the first year after the hip fracture.

No other secondary outcome was significantly different between treatment groups in our study, including mortality, preoperative waiting time, and number of patients living in a nursing home four and twelve months after surgery. Some studies have reported reduced mortality after the introduction of orthogeriatric care [8, 10–16]. The lack of effect on mortality in our study can be due to the fact that the mortality, compared to other studies, was already low before implementation of the orthogeriatric model [16].

Due to inclusion criteria, the Oslo study included more frail patients than the Trondheim study. We thus chose to include site (Oslo vs. Trondheim) as a covariate in the statistical analysis to correct for this. 

When excluding the patients admitted from a nursing home, the effect of the intervention on ADL was stronger. One possible explanation is that the frailest patients already have lost much function and the potential for reduction of further decline therefore is limited. This does not mean that these patients do not benefit of orthogeriatric care, but other instruments than the ADL scales we have used might be better to evaluate the effect (quality of life, satisfaction among patients/carers). The more fit patients in our study benefitted the most. An interpretation is that those with best function are most prone to functional decline and that optimized care therefore is particularly important in this group. A concrete strategy based on these findings would be to categorize hip fracture patients already at admission into groups based on where they realistically could be discharged (e.g. (1) Home, (2) Rehabilitation. (3) Nursing home). Tailored intervention based on these groups might be a way to optimize use of resources and at the same time secure that patients with the largest potential for rehabilitation are prioritized, a strategy in line with recommendations based on register data on hip fracture patients in Norway [45].

### Strengths and limitations

A strength of this study is the randomized controlled design of the included studies and the large sample size. Furthermore, both studies were planned in concert with future pooling of data in mind. Another strength is that patients were evaluated face to face by research nurses blinded to allocation four and twelve months after surgery. The wide inclusion criteria allowed for a heterogeneous study population and increase the generalizability of our findings. The different age distribution and differences regarding nursing home residents were accounted for by adjusting for these variables in the analyses, so we do not regard this as a limitation in the study. A limitation of the study is the lack of masking of both the patients and the staff delivering the treatment.

## Conclusions

Merged data of two RCTs conducted in Norway showed that administration of comprehensive geriatric care to hip fracture patients in an acute geriatric ward had a positive effect on I-ADL and B-ADL up to twelve months after surgery. The effect was strongest in home-dwelling patients.

## Data Availability

The data that support the findings of this study are available on request from the corresponding author.

## Abbreviations

RCTs: Randomized Controlled Trials
ADL: Activities of Daily Living
I-ADL: Instrumental Activities of Daily Living
B-ADL: Basic Activities of Daily Living
CGC: Comprehensive Geriatric Care
OC: Orthopedic Care
CGA: Comprehensive Geriatric Assessment
SPPB: Short Physical Performance Battery
NEADL: Nottingham Extended Activities of Daily Living
BADL: The Barthel ADL Index
CI: Confidence Intervals
